# Turn taking is not restricted by task specialisation but does not facilitate equality in offspring provisioning

**DOI:** 10.1038/s41598-021-01298-z

**Published:** 2021-11-08

**Authors:** Maaike Griffioen, Arne Iserbyt, Wendt Müller

**Affiliations:** Department of Biology, Behavioural Ecology and Ecophysiology Research Group, University of Antwerp, Wilrijk, Belgium

**Keywords:** Behavioural ecology, Animal behaviour

## Abstract

Sexual conflict arises when two individuals invest in their common offspring because both individuals benefit when their partner invests more. Conditional cooperation is a theoretical concept that could resolve this conflict. Here, parents are thought to motivate each other to contribute to provisioning visits by following the rules of turn taking, which results in equal and efficient investment. However, parents have other tasks besides provisioning, which might hinder taking turns. To investigate restrictions by other care tasks and whether turn taking can be used to match investment, we manipulated brooding duration in female blue tits (*Cyanistes caeruleus)* during the early nestling phase by changing nest box temperature. As expected, females subjected to cold conditions brooded longer than females under warm conditions. Yet, contrary to our prediction, females had similar visit rates in both treatments, which suggests that females in the cold treatment invested more overall. In addition, the females’ turn taking level was higher in the more demanding cold condition (and the calculated randomised turn taking levels of females did not differ), hence females don’t seem to be restricted in their turn taking strategy by other care tasks. However, males did not seem to match the females’ turn taking levels because they did not adjust their visit rates. Thus, level of turn taking was not restricted by an other sex-specific task in females and did not facilitate a greater investment by their male partners.

## Introduction

The sexual conflict over parental investment has received much research attention since the formulation of this theory by Trivers^1^. In biparental species, two unrelated individuals both benefit of their joined investment in their common brood, but they also individually pay the costs of providing this investment, which gives rise to this sexual conflict. Each parent would benefit themselves when their partner takes on a bigger share of the offspring care, so that they themselves can retain energy for self-maintenance and future reproduction^2^. One possible resolution for this conflict is to negotiate about how much each should invest. Various theoretical models have been developed in order to identify how much parental investment by each individual is expected for an evolutionary stable negotiation strategy (e.g.^3,4^). The consensus of these models is that parents should invest below the most optimal level of care and only partially compensate for a reduction in care by the partner, in order to avoid exploitation. Thus, this strategy comes with costs for the brood and hence fitness (i.e. the costs of sexual conflict and negotiation)^3,5,6^. Corresponding with the predictions of the models, the predominant outcome of previous studies indeed was partial compensation^7^. Yet, there was considerable variation between species and studies in the parental responses, ranging from no compensation to matching responses^7^. To investigate these differences, a model was designed based on the rules of the negotiation model, which incorporated the uncertainty of the brood need^8^. This model hypothesized that the variation in responses to changes in partner effort, could be explained by the amount of information the parents have about the brood (their hunger levels/need for feed)^8^. It showed that when each parent has only partial information about the brood need, they match each other’s effort when one of them raises their work rate, because that parent then serves as an informative signal of the brood need^8^.

Recently, conditional cooperation has been proposed as a strategy, which potentially can resolve the sexual conflict about investment, in such a way that equality and efficient levels of care may be achieved. It implies that parents motivate each other to invest in a common brood by providing care when their partner invests as well or withholding their investment when their partner did not contribute yet^9,10^. It resembles a tit-for-tat game in which both parents match each other’s level of investment and is named turn taking^9^. The outcome of this turn taking is an alternated pattern of visits (called alternation level), which shows the percentage that the parents alternated their feeding visits from their total investment of visits. This strategy is in line with and could potentially explain the matching responses that were found in parental investment manipulations^7,11,12^. However, it might not always be possible to take turns after the partner has invested due to limits in time because parents could be influenced or restricted by various environmental and social conditions^13–16^**,** but also by other care tasks (such as brooding and territorial defence;^17^).

Conflicting parental care tasks may interfere with turn taking, because parents are expected to only invest a certain amount of their resources into care which is equal to that of the partner in order to avoid exploitation^18^. Therefore, a change in investment in one specific parental task might cause a change in another task or even other non-parental behaviours (e.g. self-maintenance). Consequently, re-allocating more investment from one task to another by the focal parent may also cause a shifting response in the tasks of its partner. Moreover, parents can either have all parental tasks in common or tasks can be limited to one sex, which might affect how they can shift investment between those tasks. For example, in cases where both males and females might brood and feed the nestlings, the females might brood longer and feed less than the males who in turn brood less and feed more. This might affect the possibilities for strict turn taking, especially when care tasks have different costs for each of the sexes^19^. For example, a female that increases brooding (a sex-specific task in many bird species) may reduce her feeding rate in order to maintain the same overall level of investment, which is also likely to impinge on her ability/willingness to maintain her turn taking strategy of provisioning visits. As a result, this may affect the male’s feeding rate (and turn taking strategy) because the female’s turn taking level is thought to be informative for the male’s level of investment, in order to ameliorate the sexual conflict about investment^9^.

To study whether turn taking is constrained by concurrent sex specific parental care tasks in females and whether males match the females’ investment via taking turns, we used a nest box population of blue tits (*Cyanistes caeruleus*). In this species, the poikilothermic nestlings require brooding until they are 7 days old, which only the female provides by sitting on the nestlings with her brood patch which transfers her body heat to them^20,21^. The main task of the male partner is to provision during these early days of the nestling phase, so males and females don’t share all the same tasks. Our aim was to manipulate the females’ brooding behaviour by increasing or decreasing the temperature in the nest box to investigate how this affects the expression of parental feedings and the turn taking levels, which are concurrently expressed with brooding. Additionally, the reaction of the male partners to its partners’ change in time allocation in these tasks was investigated. Especially, we tested whether the males co-adjust their own behaviour in accordance. Females that are exposed to the colder temperature are expected to have longer brooding durations than females in the warm treatment and will consequently have less time to feed their offspring, which would lower their visit rate. Despite this, we expect that females exposed to cold treatments try to maintain or possibly increase their level of turn taking in order to ensure that their partner keeps feeding. When males are using the females’ turn taking levels for information about females’ investment or possibly as an indirect signal about the brood need, they will maintain visit rate in the cold treatment or even compensate for the reduction of feeding by the females. Hence, also because in this case, the reduction in females’ feeding rates results from re-allocation of investment rather than reflecting a reduced investment.

## Materials and methods

### Study species and general measurements

Experiments were conducted in a nest box population of blue tits near Antwerp, Belgium (Peerdsbos 51° 16′ N, 4° 29′ E) during the breeding season (April–May 2017). Blue tit parents provide biparental care and have various (sex specific) care tasks. Males defend the territory, feed the female during incubation and also engage in nestling provisioning, whilst females build the nest, lay and incubate the eggs, keep the nestlings warm via brooding, perform nest sanitation behaviours and provision the nestlings^22–24^. Brooding the poikilothermic nestlings by the females takes place until they are 7 days old^20,21^. The nest boxes (n = 131) were visited twice a week to check for nest building, egg laying and incubation activity. From the expected hatch date onwards, the nest was monitored daily to record the hatch day which was defined as day 0.

### Temperature manipulation

An experimental nest received either the warm or cold treatment (see below) on day 4 post hatching (sample size nests: n = 21 warm, n = 22 cold; timeframe: 23rd of April–5th of May 2017). To create equal numbers each experimental treatment was paired with an opposite treatment when possible, while taking brood mass on day 3 in consideration. While measuring brood mass on day 3, a dummy wooden box was placed on top of the nest box and a dummy bag was placed inside the box, on the tree side. In the morning of the experiment, the bag was filled with the device of either the hot (electronic handwarmers) or cold (frozen ice element) treatment and the wooden box on top of the nest box was replaced by a similar wooden box filled with camera equipment. A small Ibutton (I-wire DS1922L; interval rate: 60 s; logging: 0.5 °C) in the corner of the nest box registered the temperature. The difference between the warm and cold treatment was 1.2 degrees of Celsius (warm mean [min–max]: 12.8 °C [5.5–22.5]; cold mean [min–max]: 11.6 °C [5.0–22.0]) The infrared camera (420TVL; Pakatak PAK-MIR5, Essex, UK) itself was placed under the lid facing down on the nest to record parental behaviour. The treatment started between 8 and 10 AM (Mean 09:06 AM, min–max [08:01–09:59 AM]) to avoid the colder temperatures early in the morning, which could interfere with the effects of the treatments. The temperature manipulation was kept to a minimum duration for welfare reasons and technical limits (2 h maximum).

### Behavioural measurements

From the video recordings, the first half hour was discarded to reduce the influence of human disturbance on the parental behaviours and to let the temperature manipulation settle in. The subsequent hour of the video recording was scored with the program of ObserverXT (version 10.5.572, 2011, Noldus Information Technology, Wageningen, The Netherlands; all videos are scored by M. Griffioen). Only 1 h of parental behaviours was scored, because previous research has confirmed that this is sufficient to acquire reliable measures^25,26^. By analysing a short time period, possible environmental changes during the day are kept to a minimum^14,27,28^. Males and females were distinguished by their PIT tag (from previous years which can be on different sides of the legs and/or colour) or by their head pattern (males have darker stripes on their head). For both sexes the number of feeding visits were scored and for the females additionally the brooding duration. The total duration of brooding was calculated by summing all brooding bouts of the female. After video scoring, only the nests that were biparental (i.e., where both male and female fed at least once) were used for further statistical analysis (sample size: n = 18 warm, n = 17 cold). Visit rates were calculated as visits per hour for both parents separately. Male and female turn taking rates were calculated from the visit sequences with an approach that is comparable to a Markov analysis^9,29^. This calculation implements both the nature of the visit (alternated or not) and the duration of the inter-visit intervals, which are divided to acquire a λ (rate of following the partner) and μ (rate of following itself) (as in^9,29^). Dividing λ by μ provides an estimate for a turn taking level (λ/μ; turn taking > 1 indicates that the individual follows its partner more than itself). To investigate the effects of the visiting pattern (e.g. refractory period^9^) and the stochastic effects of the environment at the time of the experiment^9,14,27^, we used randomisations of the visit sequences to calculate randomised values of turn taking. Randomisations of turn taking were made by randomly order the visit sequence of each nest a 1000 times^9,13^. For these 1000 times the turn taking level of the male and female was calculated with the mathematics explained above. Comparing the randomised turn taking values per sex between the treatments will reveal whether the parents’ observed (real) turn taking values are affected differently per treatment by other factors (e.g. predator disturbance, weather influences^13,14,27^).

### Compliance with ethical standards and data availability

Animal use and experimental design were approved by the Ethical Committee of the University of Antwerp, Belgium (ID: 2015–64). The experiment was carried out in accordance with the guidelines of FELASA (Federation of European Laboratory Animal Science Associations). The dataset supporting this article comes as supplementary material.

### Statistical analysis

To investigate the effect of our treatment on the brooding behaviour of the females, we performed a linear model analysis. Julian date (date of the experiment) and brood mass on day 3 were used as covariates to correct for the brood value, seasonal changes in food availability and ambient temperature. Next, the behavioural response of the females towards the treatment and the reaction of the males towards their partner was investigated separately in order to test the specific hypotheses that we had for each sex. Hence, for females a linear model was used to test the effect of treatment on visit rate. We tested treatment as fixed effect and included brood mass (on day 3) and Julian date as covariates. The response variable was log transformed to acquire normally distributed residuals. For males we used a similar linear model, in which we tested for treatment effects on visit rate. This analysis included Julian date and brood mass as covariates. Additionally, to test whether the male’s response was affected by the female’s behaviour, the male model was repeated twice with either female brooding or female visit rate incorporated. For testing the effects on the observed turn taking levels we again used separate linear models for each sex. For both male and female observed turn taking values we performed separate linear models with treatment as fixed effect, and brood mass (on day 3) and Julian date were included as covariates. For the expected turn taking values, two models were made for each sex, containing treatment as fixed effect and Julian date and brood mass as covariates.

We used the Shapiro normality test to investigate whether the residuals of the models were normally distributed (normal distribution assumed at W > 0.90) and also visually checked QQ-plots of the residuals. The significance of the fixed factors was investigated using model selection with the drop1 function and with a critical α level of 0.05. The analyses were performed in the statistical program R studio (version 1.1.423 and R version 3.4.3, R core Team, 2018).

## Results

### Brooding duration and visit rate of females

**Figure 1:**
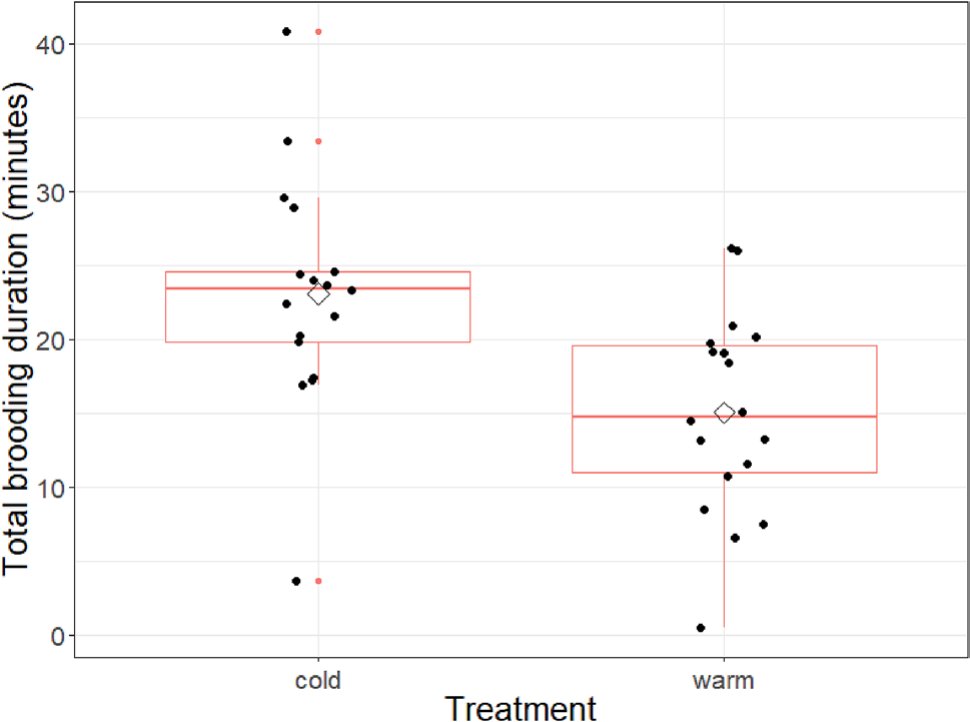
Difference in total amount of time spent brooding by the female, separated by treatment. Boxplot distribution (red), mean (diamond) and data points (black) are presented and represent the time a female is brooding within one-hour of treatment in either warm or cold conditions. Mean ± SE of cold treatment: 23 ± 2 min; warm treatment: 15 ± 1.6 min.

**Figure 2:**
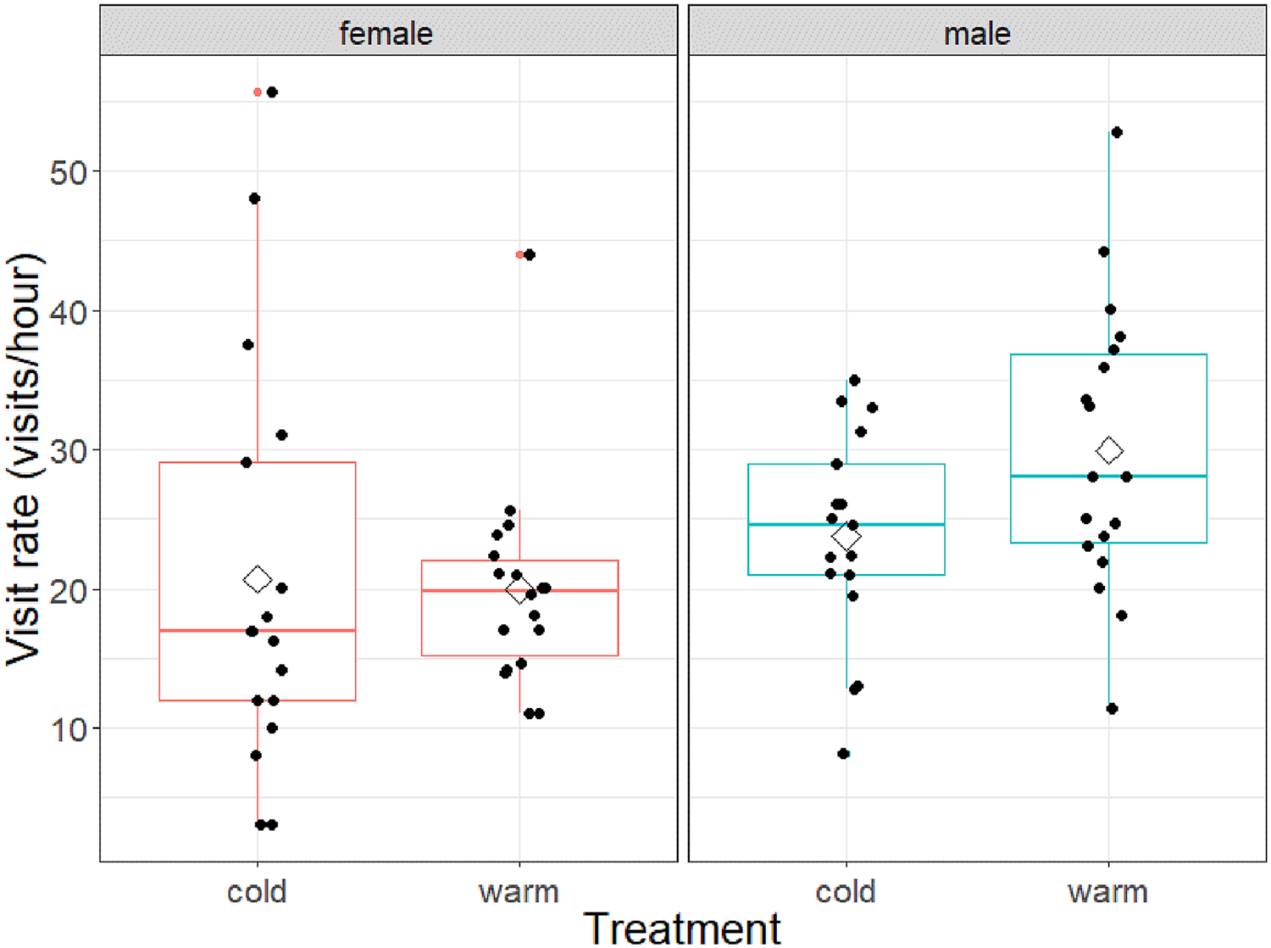
The effect of temperature manipulation on parental nest visit rates (number of visits/hour) for both female and male parents when nestlings are 4 days old. Boxplot distribution (red), mean (diamond) and data points (black) are presented. Females and males were analysed separately. Mean ± SE for females of cold treatment: 20.7 ± 3.6 Visits per hour; females of warm treatment: 20 ± 1.8 visits/hour; males of cold treatment: 23.7 ± 1.8 visits/hour; males of warm treatment: 30 ± 2.4 visits/hour.

The total time spent brooding by the females differed between the cold and warm treatment (Treatment: F_1,32_ = 15.9, P = 0.0004; cold treatment mean ± SE 23.2 min ± 1.55 min, n = 17; warm treatment 15.2 min ± 1.37 min, n = 18; see Fig. 1). This strongly indicates that the manipulation of nest box temperatures influenced the brooding duration of females. During the cold treatment females spent about 53 percent more time brooding than females in the warm treatment. Julian date did not influence the time females spent on the nest (Julian date: F_1,31_ = 0.006, P = 0.94). However, heavier broods were less brooded by females (Total brood mass on day 3: estimate = -0.56, SE = 0.22, F_1,32_ = 6.66, P = 0.015). The visit rates did not differ between females exposed to the warm or cold treatment (Visit rate: F_1,32_ = 1.83, P = 0.19; see Fig. 2). Neither of the covariates had a significant effect on the female’s feeding rate (Julian date: F_1,33_ = 1.77, P = 0.19; total brood mass: F_1,33_ = 2.82, P = 0.10).

### Males’ visit rate

The visit rates of the males tended to differ between the treatments with males in the cold treatment tending to have lower visit rates than males in the warm treatment (Treatment: F_1,33_ = 4.10, P = 0.051; see Fig. 2; Julian date: F_1,31_ = 0.005, P = 0.95; and brood mass: F_1,32_ = 0.29, P = 0.59). When testing for the influence of female behaviours, neither brooding nor provisioning by the female affected the male provisioning behaviour significantly (Female brooding: F_1,32_ = 0.46, P = 0.502; female provisioning: F_1,31_ = 0.04, P = 0.84).

### Turn taking of females and males

**Figure 3:**
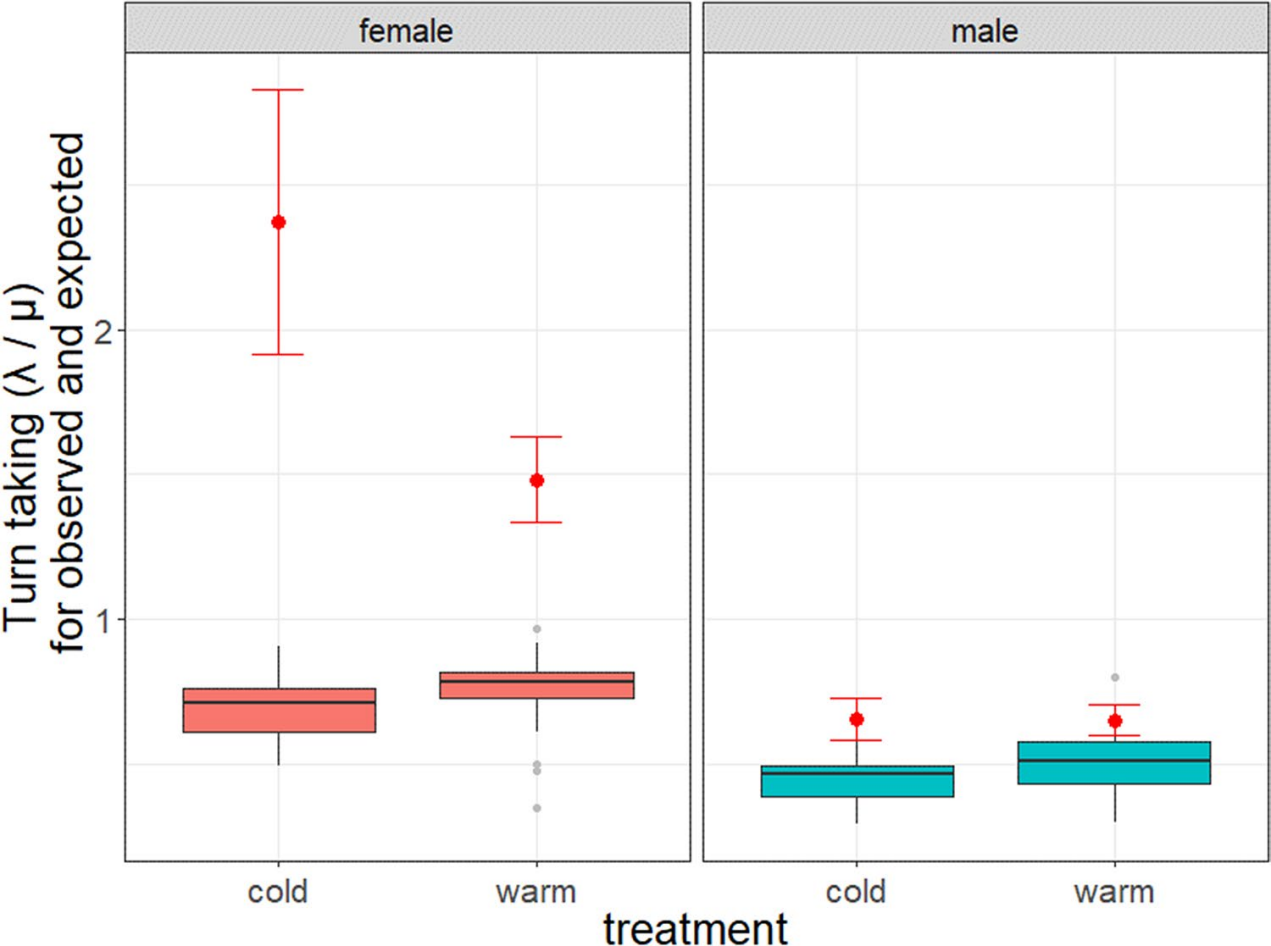
The turn taking levels when nestlings are 4 days old, separated for treatment and sex. Mean ± SE are represented for the observed turn taking values (red points) both females and males for the cold and warm treatment. Boxplot distributions of the expected/random turn taking levels is presented for the sexes and treatment (based on 1000 randomisations of the visit sequence). Turn taking as λ/μ; turn taking > 1 indicates that the individual follows its partner more than itself. Observed turn taking (λ/μ) mean ± SE cold treatment females: 2.4 ± 0.45; warm treatment females: 1.5 ± 0.15; cold treatment males: 0.65 ± 0.07; warm treatment males: 0.65 ± 0.005. Expected turn taking mean ± SE cold treatment females: 0.69 ± 0.03; warm treatment females: 0.74 ± 0.04; cold treatment males: 0.45 ± 0.02; warm treatment males: 0.74 ± 0.04.

The expected (randomised) turn taking values of females in the cold and warm treatment did not differ (Treatment: F_1,33_ = 1.02, P = 0.321). Neither Julian date nor brood mass significantly affected the randomised turn taking levels (Julian date: F_1,31_ = 0.018, P = 0.896; brood mass: F_1,32_ = 0.60, P = 0.445). Furthermore, females exposed to the cold treatment had significantly higher observed turn taking levels than females from the warm treatment (Treatment: F_1,32_ = 6.08, P = 0.019; see Fig. 3). Julian date did not influence the observed turn taking level of the females (Julian date: F_1,31_ = 0.014, P = 0.905), but brood mass did, with lower turn taking levels of the females with heavier broods (Brood mass: estimate = -0.091, SE = 0.042 F_1,32_ = 4.68, P = 0.038). For males, the expected turn taking level showed a trend, with randomised values of the warm treatment showing slightly higher values (Treatment: F_1,33_ = 3.59, P = 0.067). The Julian date and brood mass did not affect the expected turn taking levels of males (Julian date: F_1,31_ = 1.36, P = 0.25; brood mass: F_1,32_ = 2.0, P = 0.17). The observed turn taking levels of the males did not differ between treatments (Treatment: F_1,31_ = 0.0009, P = 0.977; see Fig. 3). Julian date and brood mass had both no significant effect on the male turn taking levels (Julian date: F_1,33_ = 1.83, P = 0.185; brood mass: F_1,32_ = 0.16, P = 0.694).

## Discussion

It has been hypothesized that turn taking during offspring provisioning allows parents to match each other’s level of investment, yet it might not always be possible to feed after the partner has fed because parents might have other, potentially concurrent sex-specific parental care tasks as well. To test whether conflicting parental care tasks impinge on turn taking, we applied a temperature manipulation during the early nestling period to change the females’ brooding duration. Females exposed to the cold treatment spent more time brooding than females in the warm treatment. However, even though these females invested extra time and possibly extra energy in brooding, they fed their offspring at similar levels as females in the warm treatment. Turn taking levels of females in the cold treatment were higher than those of females in the warm treatment, which was expected to stimulate the male to enhance offspring provisioning. Yet the male partners neither responded to the increased workload, nor to the higher turn taking levels, on the contrary, they even seemed to feed less.

### Females are responsive to the brood need

In line with the objective of our manipulation, blue tit females responded to the treatment with longer brooding duration in the cold treatment compared to the warm treatment. This is to our knowledge the first experimental evidence that shows that females have different brooding durations during the nestling stage in response to the temperatures in the nest box. Females should respond to differences in temperature, because the nestlings at this stage (day 4 post hatching) are still poikilothermic and thus rely on the brooding behaviour of their mother^20,21,30^. Considering that in our study species, females are the only sex that broods the nestlings and therefore also spend more time on the nest than males, it seems obvious that they are more aware of the need of the brood and will respond to changes in need^8,31,32^ (see below).

There is some evidence that in populations of female orange crowned warblers (*Oreothlypis celata*) the brooding duration varies with geographical temperature differences. Females spend more time brooding in Northern and colder situated populations compared to more Southern warmer populations^33^. Within the northern and southern populations, however, the ambient temperature did not influence the brooding behaviour of the females. In our experiment we did not change the ambient temperature, but only the temperature inside the nest box, which appears to be a sufficient trigger to adjust their brooding behaviour. In line with this, Amininasab et al. found that when ambient temperature was high, nest temperature increased and females reduced the incubation time of their eggs, exchanging it for time off the nest for self-maintenance activities^34^. While this research focused on the incubation stage instead of the nestling stage, it does suggest that females are responsive to environmental temperature changes. Incubation of eggs and brooding offspring are similar in the demand of heat transfer, both come with energetic costs (see^35^). As Amininasab et al. showed that when females did not have to incubate as long due to higher temperatures, they invested their time in self-maintenance and so, females seem to be restrained by their energy reserves under certain environmental conditions^34^.

### Females’ extra effort

Females exposed to the cold treatment had higher brooding durations, but intriguingly they had similar visit rates as the females of the warm treatment, so they seem to work harder. This is contrary to what we had expected, as we hypothesized differences in brooding duration would be reflected in other parental tasks such as their visit rate due to energy and/or time restrictions and in order to maintain the same level of investment^17,18^. It is likely that females lowered the time they spent on self-maintenance and self-feeding in order to facilitate their greater investment^34^, which then still represents a cost for the females in terms of reduced survival and thus reduced future reproduction^2^. However, this was not measured here. It is also possible that the brooding of nestlings is energetically less demanding so that the costs of additional brooding might be lower than we expected (^27^, but see ^28^). Another explanation might be that the females compensate later, e.g. in a different stage of the reproductive event^38^. Several studies have indeed shown evidence for these carry-over effects from one stage to another later stage^39,40^. Furthermore, females might have changed the prey sizes in order to save time (see ^41,42^) to be able to have a high visit rate. However, previous work showed that prey sizes had limited effects as such that parents did not change it to their own benefit^26,29^. Considering all these possible options, the females in our experiment seem to be capable of adjusting their investment according to the present context in order to maintain the same overall level of investment.

Females from the warm treatment were relieved of their brooding duty, but had lower turn taking levels as compared to the females from the cold treatment. Hence, it seems that turn taking was not restricted by other parental tasks (brooding in this case). Yet, turn taking might have been of less relevance for these females because the nestlings did not need extra (brooding) care. Furthermore, the higher turn taking levels from females of the cold treatment suggest that it could be relevant for the coordination of parental care and might inform males about the extra investment of the females. Females seem to show higher turn taking levels, which might also come with additional costs as monitoring their partner might take more time ^26^. Yet here, females spend more time on the nest brooding which could make it easier to monitor her partners’ investment (visits to the nest) (see also^43^), so monitoring their partner might be less time consuming. Brooding females might have had a higher monitoring accuracy which might facilitate their high turn taking. However, they also had high visit rates which contradicts the predictions of the models from Johnstone and Savage that showed that the parent with high monitoring accuracy, had lower turn taking and visit rates compared with its less informed partner^44^. This difference might be due to that in our case the more informed females were subjected to an experiment which changed the brooding demand of the nestlings, which could alter the expected behavioural response of the females. This forced the females to invest more and thus turn taking might be a way in which females could inform their male partners about the extra need and/or their extra investment, which will be discussed in the next section.

### Males do not respond to females

While females in the cold treatment seem to put more investment in the current reproductive event, males did not seem to change their care. The high turn taking level of females did not elicit a response by the males to feed more. An explanation might be that females stop brooding and leave the nest when the males enter the nest box. So, the males will encounter the females on the nest when they come to feed. This might give a signal to the males that the females are investing less into care because she was not outside looking for prey. Especially, since the male might not be capable to detect the signs of need for extra brooding of the nestlings, as brooding is a sex-specific task. Females are therefore spending more time on the nest and could gather more information, leaving the male parents to be less informed or reliant on the females for information^8,31^. If there is such an asymmetry in the information about the brood need, it has been hypothesized that the better-informed parent should, besides working harder, also react stronger to changes in brood need. The partner that has less information is predicted to show a weaker compensation or no compensation at all to the increased investment of the informed parent^8^. In our case, the less informed males even tended to invest less in provisioning in the situation where females invest more due to the cold treatment. In terms of partner equality, males should invest more into care to keep it evenly distributed and could use turn taking to match in effort. However, the males do not seem to respond to the high turn taking levels of their female partners, so maybe they use a certain level of turn taking as a signal to inform partners that they are still investing in order to maintain their pair bond^26^. The duration of the manipulation might have been too short for the males, even though the females did respond to our short-term temperature manipulation. However, turn taking predicts immediate responses of the parents to changes of their partner. So, despite the short duration of the temperature manipulation, males should have reacted by matching their partners’ investment. Additionally, the males’ observed turn taking rates are below 1, indicating that males are visiting the nest more often after a visit by themselves (so not follow the visits of the females). The males’ expected turn taking values also tended to differ, indicating that in both treatments the observed turn taking values might be affected differently by other stochastic or environmental factors (e.g. refractory period, predators^9,14,27,28^). Either way turn taking does not seem to function here (for males) to facilitate strict equality in parental care.

### Turn taking as a mechanism to resolve conflict

Previous work has shown that overall, partial compensation is how parents react to a change in their partners’ effort^7^. However, matching responses were also found^7,11^, and have been tested to explain their occurrence and stability^8,9,45^. Turn taking, as a strategy to resolve conflict, is a strategy which allows parents to match each other’s investment. Whether a species is more likely to cooperate or partially compensate will depend on a lot of factors (e.g.: environment, life history traits of a species, individual status such as age, social environment^46–48^) and thus species might also vary along a continuum from being in conflict and cooperating^46,47^. In line with this, Lejeune et al. found that in a population of blue tits, the ecological differences along an altitudinal gradient affected the pair coordination in synchrony and alternation^49^. House wrens (*Troglodytes aedon*) were also found to be affected by their environment in their coordination, with pairs form rural sites having more synchronisation and alternation than those form suburban sites^50^. Another example is in the same blue tit population, which is used here, a sex-specific partial compensation was observed in response to a temporal mate removal experiment, but a clear matched response followed upon the reunion of the pair^31^. Our current results showed that female blue tits also seem to adjust their investment regarding the brood need of the nestlings, in terms of brooding and turn taking. Males on the other hand, did not seem to change their investment in accordance with the brood need or the behaviour of their partner, indicating that there might be sex differences in detectability and responses. Indeed, females are the best informed sex in this case, because they spent more time on the nest for brooding, which is in line with the prediction that the best informed parent should respond to changes in need^8^.

Coordinated feeding visits (turn taking and alternation) have been found in a number of observational studies (Great tit *Parus major*^9,15^; Blue tit *Cyanistes caeruleus*^26,29,31^*;* Long-tailed tits *Aegithalos caudatus*^51^; Canaries *Serinus canaria*^17^; Acorn woodpecker *Melanerpes formicivorus*^52^; Blackcap *Sylvia atricapilla*^53^; Chestnut-crowned babbler *Pomatostomus ruficeps*^13^; Manx shearwater *Puffinus puffinus*^54^; Little auk *Alle alle*^55^). Multiple studies have also shown that parents do take turns or show alternated patterns that are higher by chance^9,13,15,17,51,52^, but see^14,27,28^. Our study is the first to show that despite a manipulation, the expected randomisations did not change for females. Despite that this does not show whether parents ‘actively’ take turns, it does suggest that in both treatments turn taking is shaped by similar amounts of chance. However, in the warm treatment, males tended to have higher expected turn taking values, which is in line with the higher males’ visit rates that drive the expected values. Although we cannot completely rule out that common environmental conditions are driving our results (and thus whether parents ‘actively’ take turns), it is still interesting that the parents show different responses.

## Conclusion

Blue tit females were responsive to an experimentally increased brood need, in which they are investing more in care, which will likely come at a cost. Moreover, the turn taking level of females did not seem to be restricted by an increase in brooding. Brooding might actually facilitate turn taking by making it easier for the female to monitor her partners’ visits. The high turn taking levels of females that had to invest more suggests that it might be important for the coordination of parental care. However, regardless of the extra effort in terms of visits and turn taking by females from the cold treatment, males did not respond accordingly. This raises doubts as to whether turn taking acts as a mechanism which ensures equal investment levels to resolve conflict over care. However, males might not respond or are not motivated to feed because they encounter the female more often on the nest which could give a negative signal about the females’ investment. By not sharing the burden with their females, the males do not fully cooperate with their partners, at least in the time frame of our study. Thus, turn taking was not restricted by other sex-specific task in females but did not facilitate more investment by their male partners.

## Supplementary Information

Supplementary Information 1.

Supplementary Information 2.
